# Investigation on the Attitudes and Perspectives of Medical and Nursing Staff About Euthanasia: Data From Four Regional Greek Hospitals

**DOI:** 10.7759/cureus.53990

**Published:** 2024-02-10

**Authors:** Pantelis Stergiannis, Effrosyni Fanouraki-Stavrakaki, Panagiota Manthou, George Intas

**Affiliations:** 1 Faculty of Nursing, National and Kapodistrian University of Athens, Athens, GRC; 2 Surgical-Orthopedic Clinic, General Hospital Health Center of Sitia, Sitia, GRC; 3 Infection Prevention, University of West Attica, Athens, GRC; 4 Nursing, General Hospital of Nikaia, Athens, GRC

**Keywords:** perspectives on euthanasia, attitudes towards death., medical-nursing staff, ethical dilemmas, euthanasia

## Abstract

Introduction: The good and benefit of the patient are the main drivers of the decisions that health professionals are asked to make. However, the definition of the good and the actions required for benefit are not always simple and self-evident. The intractable ethical dilemma of euthanasia has been the subject of extensive debates over the years, and numerous studies have been carried out in an attempt to record the attitudes and opinions of both health professionals and the general population.

Method. This research aims to investigate the opinions and perspectives of the medical and nursing staff of the four regional hospitals regarding euthanasia and to detect the factors that advocate for and against it. Two hundred and eighteen medical and nursing staff members from four regional hospitals in Lasithi participated in the research, whose opinions and influencing factors were investigated using a questionnaire consisting of four sections. The first included demographic and general characteristics questions; the second was the Euthanasia Attitude Scale (EAS); the third was the Death Attitude Profile-Revised (DAP-R); and the last was the Daily Spiritual Experience Scale (DSES). The SPSS software version 25.0 (IBM Corp., Armonk, NY) was used to analyse the data.

Results: Of the total, 78.0% of the participants were women, with an average sample age of 44.5 years. 65.1% were married, 23.4% were physicians, while 76.6% were nurses. The mean Euthanasia Attitude score (70.89) is moderate, ranging from 30 to 120, with higher scores suggesting more favourable sentiments. Euthanasia was viewed positively by 24.3% of respondents. There was no significant difference in positive attitudes between medical and nursing staff. However, the nursing staff had significantly lower average levels of General Orientation for Euthanasia, for the Role of Healthcare Professionals in Euthanasia, Values & Ethics, or Daily Spiritual Experience, and conversely higher levels of scores on Patients' Rights Issues for Euthanasia or Death Acceptance.

Conclusions: Health professionals were found to have moderate attitudes about euthanasia, with no significant difference between them, as well as moderate degrees of death and everyday spiritual experience. Overall, a more favourable euthanasia attitude was shown to be strongly associated with individuals who were single, divorced, or widowed, with less death acceptance or more neutral acceptance, but not with daily spiritual experience.

## Introduction

Euthanasia, the painless inducement of a quick death, is a complex ethical issue due to its complexities and the challenges it presents. With the aging population and chronic diseases, advances in science have led to longer patient survival times, but this doesn't necessarily improve the quality of life [1]. When the path to death appears to be extremely difficult, dying is frequently regarded as the least terrible or even soothing option. The relevance of "euthanasia" is evident, as it, along with life extension, is one of the primary research topics in current bioethics [2]. There are various concerns highlighted, the most significant of which is whether a person with a fatal illness who wishes to die should be kept alive at all costs. It is critical to find a legislative solution to the dilemma in order to provide individuals with the right to decide whether and how to end their lives in specific circumstances [3].

Morals, religious beliefs, social and cultural roots, technological and medical developments, and the possibility of exploitation are all reasons for concern [4]. Various interest groups and organisations have produced 62 statements or advocacy documents in support and opposition to euthanasia and assisted suicide, with the goal of exerting political influence and promoting reform [5]. These declarations represent the global discussion about end-of-life concerns. Only a few nations have legalised or decriminalised euthanasia and other medically assisted suicides, with particular restrictions. Currently, only four European Union nations have legalized active and medically assisted suicide: the Netherlands, Belgium, Luxembourg, and Spain, most recently [6]. In Greek reality, euthanasia is prohibited due to a lack of specific laws on the issue, and the act's illegality is defined by the Criminal Code's basic principles that ban voluntary homicide [7,8].

This research paper investigates the opinions and attitudes of medical and nursing staff in four public nursing units regarding euthanasia, focusing on demographic characteristics, work profile, attitude towards death, and religiosity to identify factors advocating for or against euthanasia, considering legal, religious, and medical dimensions.

## Materials and methods

A cross-sectional observational study was conducted between February and April 2023 with 218 health professionals, including medical and nursing staff from four regional Greek hospitals. The medical and nursing staff who participated in the research were fully informed about the purpose of the study and given written consent, which they could withdraw at any time. Out of 502 registered patients at the hospitals, 447 were on duty, with 218 (51% of personnel) responding.

Inclusion and exclusion criteria

The study's inclusion criteria comprised physicians and nurses, independent of duration of service, past experience, or kind of position (permanent, auxiliary, etc.). Physicians and nurses on prolonged leave (maternity, unpaid leave, in-service training, etc.), as well as those on relocation for other postings, were unable to participate, so they were excluded from the study.

A validated and reliable questionnaire was used to collect data. The first part included socio-demographic variables and factors. The second segment includes 30 statements from the Euthanasia Attitude Scale (EAS) [9]. The third component consists of 32 statements about attitudes towards death from the Death Attitude Profile-Revised (DAP-R) scale [10]. The final section includes 16 sentences from the Daily Spiritual Experience Scale (DSES), which assesses the research participants' religion and spirituality [11]. The scales' previous use, validity, and methodological structure were all clearly defined in the required bibliographic references, which were obtained via the researcher's personal electronic correspondence with the scales' creators (DSES) and Greek scale creators.

The 7th Health Region Scientific Council approved research at four hospitals: Ierapetra (57/6-3-23), Sitia (641/02-02-2023), Neapoli (3/20-01-2023), and Aghios Nikolaos (70/07-12-2022) in accordance with the Code of Ethics and Ethics. The research's purpose was fully disclosed to the medical and nursing staff who took part in it, and they were given written consent.

Statistical analysis

The thesis research data was analysed using the SPSS software version 25.0 (IBM Corp., Armonk, NY). The reliability of the three scales-the Daily Spiritual Experience Scale (DSES), the Euthanasia Attitude Scale (EAS), and the Death Attitude Profile-Revised (DAP-R)-was evaluated using the Cronbach technique. Significance levels are two-sided and statistical significance was set at 0.05.

## Results

Of the total, 78.0% of the participants from the four hospitals in the Lasithi region were females. The average age of the medical and nursing staff was 44.5 years, and 65.1% were married. In terms of education, 39.0% were nursing assistants and 61% were nurses, whereas 11.9% had master's degrees and 1.4% held PhDs (Table 1).

**Table 1:** Demographic data MSc: Masters in Science; PhD: Doctor of Philosophy; n: Number of participants

Parameters	Demography	n	%
Sex	Men/women	48/170	22.0/78.0
Age, years	Mean age ± standard deviation (range)	44.5±10.6 (20-67)	
Marital status	Not married	76	34.9
	Married	142	65.1
Education	Nurse assistants	85	39
	Nurse	133	61
Degree	MSc	26	11.9
	PhD	3	1.4

According to Table 2, 23.4% of the 218 participants in the current study were doctors, and 76.6% were nurses. 17.6% of doctors were general medicine physicians, with 23.5% having worked for less than five years and 11.1% for more than thirty years (Table 2).

**Table 2:** Working data of health staff

Parameters	Profession	n	%
Job	Medical Staff	51	23.4
	Nursing Staff	167	76.6
Physician Speciality	General Medicine	9	17.6
	General Surgery	9	17.6
	Agricultural (without)	8	15.7
	Biopathology	4	7.8
	Pathologist	4	7.8
	Pediatrician	4	7.8
	Anesthesiologist	3	5.9
	Cardiologist	3	5.9
	Orthopedic	3	5.9
	Gynecologist	1	2.0
	Country Doctor	1	2.0
	Nephrologist	1	2.0
	Urologist	1	2.0
Years of Work	0-5	51	23.5
	5-10	18	8.3
	10-15	20	9.2
	15-20	31	14.3
	20-25	37	17.1
	25-30	36	16.6
	>30	24	11.1

Table 3 displays the scores of the research participants on the Daily Spiritual Experience Scale (DSES), Death Attitude Profile-Revised (DAP-R), and Euthanasia Attitude Scale (EAS). The average euthanasia attitude score (70.89) ranges from 30 to 120, with higher values indicating more positive sentiments; therefore, it is considered moderate. Similarly, in Attitude Towards Death and likely ranges of 1-7, death avoidance had considerably higher mean scores (and moderate to high levels) than lower neutral acceptance (p<0.001) (Table 3). Less than half of the participants, or 24.3%, had positive attitudes towards euthanasia. It is noted that between medical and nursing staff, there was no significant difference in the distribution of positive attitudes (21.6% versus 25.1%, respectively, p = 0.602).

**Table 3:** Score levels of the research participants. The scores were obtained on Euthanasia Attitude Scale (EAS), Death Attitude Profile-Revised (DAP-R), and The Daily Spiritual Experience Scale (DSES).

Score	Average	Typ. excl.	Median	Min	Max	Asymmetry
Euthanasia position (EAS)	70.89	7.36	71.50	42.00	112.00	0.18
General euthanasia orientation	33.13	3.90	33.00	15.00	52.00	-0.43
Patients' rights issues	14.99	4.59	14.00	2.00	28.00	0.25
Role of technology in sustaining life	12.32	2.20	12.00	5.00	20.00	0.23
Role of health professionals	10.11	2.07	10.00	4.00	16.00	0.00
Values & ethics	11.53	3.02	11.00	5.00	20.00	0.26
Death Attitude Survey (DAP-R)						
Mean escape	4.04	0.43	4.00	2.20	5.20	-0.20
Avoiding death	4.12	0.57	4.20	2.00	5.80	-0.15
Acceptance of death	4.09	1.20	4.00	1.00	7.00	-0.15
Fear of death	3.60	0.68	3.43	2.00	5.57	0.57
Neutral acceptance	2.56	0.87	2.40	1.00	7.00	1.14
Daily Spiritual Experience (DSES)	53.71	16.03	53.00	19.00	91.00	-0.08

Table 4 displays the approximate correlations of scores between the three scales. Positive attitudes towards euthanasia correlate with lower acceptance of death (p <0.05) or neutral acceptance, but not with daily spiritual experiences (p <0.05). Daily spiritual experience has a significant correlation with general euthanasia orientation, higher values and ethics (p <0.05), lower acceptance of death (p <0.05), and the increased role of healthcare professionals in euthanasia (p <0.05) (Table 4).

**Table 4:** Correlation of Attitude to Euthanasia, Attitude to Death Investigation and Daily Spiritual Experience scores of research participants. ^a^A higher score indicates more positive attitudes towards Euthanasia; ^b^A higher score indicates a higher degree of the concept of each subscale; ^c^A higher score indicates spiritual experiences that the person may have during the day; * p-value<0.05.

	1	2		3	4	5	6	7	8	9	10	11
Scale	5 r-Pearson											
1. Attitude of Euthanasia^a^	--											
2. General orientation toward euthanasia	0.847*											
3. Patients' rights issues	0.431*		0.129									
4. Role of technology in sustaining life	0.659*		0.345*	0.612*								
5. Role of health professionals	0.390*		0.375*	-0.418*	-0.063							
6. Values & ethics	0.326*		0.486*	-0.526*	-0.185*	0.585*						
Investigating Attitudes Towards Death ^b^												
7. Mean of escape	-0.047		-0.026	-0.147*	0.019	0.097	0.023					
8. Avoidance of death	-0.075		-0.018	0.039	-0.062	-0.154*	-0.060	-0.110				
9. Acceptance of death	-0.265*		-0.229*	0.095	-0.185*	-0.294*	-0.236*	-0.178*	0.172*			
10. Fear of death	0.088		0.064	-0.040	0.044	0.036	0.160*	-0.115	0.009	-0.091		
11. Neutral acceptance	0.255*		0.188*	0.136*	0.150*	0.063	0.135*	-0.087	0.071	-0.108	0.024	
12. Daily Spiritual Experience ^c^	0.079		0.188*	-0.273*	-0.158*	0.285*	0.343*	0.107	-0.022	-0.475*	-0.015	0.056

Women have a higher acceptance of death (p <0.05), a lower daily spiritual experience (p <0.05), and a lower agreement in values and ethics about death (p <0.05). Married individuals reported poorer daily spiritual experiences (p <0.05) and a smaller role for health professionals in euthanasia (p <0.05). However, participants' higher educational levels appear to be strongly associated to a greater role for health professionals in euthanasia, values and ethics, a lower acceptance of death, or a higher daily spiritual experience (p <0.05) (Table 5).

**Table 5:** Correlation of the scores of Attitude to Euthanasia, Investigation of Attitude Towards Death and Daily Spiritual Experience of the research participants with their special characteristics. ^a^A higher score indicates more positive attitudes towards Euthanasia; ^b^A higher score indicates a higher degree of the concept of each subscale; ^c^A higher score indicates spiritual experiences that the person may have during the day; * p-value<0.05.

	Sex (1:men, 2:women)	Age(years)	Marrital Status (1: single/ divorced/widowed. 2: married)	Education Level	Years of working experience (per 5-year change)
Scale	5 r-Pearson				
1. Attitude of Euthanasia a	-0.037	-0.022	-0.124	0.088	-0.051
2. General guidance on euthanasia	-0.127	-0.011	-0.166*	0.149*	-0.044
3. Patients' rights issues	0.154*	-0.049	0.095	-0.193*	-0.055
4. Role of Technology in preserving life	0.046	-0.092	-0.009	-0.020	-0.082
5. Role of Health Professionals	-0.015	-0.019	-0.152*	0.155*	-0.016
6. Values & Ethics	-0.150*	0.088	-0.099	0.226*	0.048
Investigating Attitudes Towards Death b					
7. Means of Escape	0.011	0.062	0.033	0.025	0.053
8. Avoiding death	0.054	-0.059	-0.007	-0.083	-0.077
9. Acceptance of death	0.181*	0.001	0.090	-0.172*	-0.008
10. Fear of death	-0.118	-0.048	-0.081	0.023	-0.071
11. Neutral acceptance	0.093	0.087	0.085	0.050	0.069
12. Daily Spiritual Experience c	-0.297*	0.064	-0.138*	0.318*	-0.019

The death attitude survey, daily spiritual experience, and participant characteristics are all shown in Table 6 as multiple linear regressions of euthanasia attitudes; nevertheless, no particular connections are found. Two regression models were utilised to explain the variance (addition) in everyday spiritual experiences. The second model reveals that, although the participants' current relationships remain relatively unchanged, the more positive attitude towards euthanasia is significantly correlated with the participants' family status, particularly for those who are single, divorced, or widowed and have lower death acceptance (p =0.001) or higher neutral acceptance (p <0.001). There was no significant relationship found between euthanasia views and everyday spiritual experiences (p >0.05) (Table 6).

**Table 6:** Multiple Linear Regression of Euthanasia Attitude on Participant Characteristics and Survey Participants' Death Attitudes & Daily Spiritual Experience. ^a^A higher score indicates a higher degree of each subscale concept; ^b^A higher score indicates spiritual experiences that the person may have during the day.

		Euthanasia Attitude (high score " more positive attitudes)							
		1st model				2nd model			
		β	95%ΔΕ		p-value	β	95%ΔΕ		p-value
Gender (1: men, 2: women)		Marital status (1: single. divorced. widowed. 2: married)	-2.60	2.20	0.872	-0.39	-2.83	2.04	0.751
Education (1: DE. 2: TE. 3: PE)		Job (1: Doctors. 2: Nurses)	-4.16	-0.21	0.030	-2.24	-4.22	-0.26	0.027
Death Attitude Investigation (DAP-R) a Means of Escape		Avoiding death	-1.83	1.90	0.969	0.10	-1.77	1.97	0.916
Acceptance of death		Fear of death	-3.35	3.79	0.903	0.06	-3.53	3.64	0.976
Neutral acceptance Gender (1: men, 2: women) Education (1: DE. 2: TE. 3: PE)	Daily Spiritual Experience (DSES) b	-1.42	-3.61	0.77	0.203	-1.37	-3.56	0.82	0.219
	Marital status (1: single. divorced. widowed. 2: married)	-0.72	-2.35	0.91	0.386	-0.64	-2.27	1.00	0.443
	Job (1: Doctors. 2: Nurses)	-1.30	-2.12	-0.48	0.002	-1.48	-2.38	-0.58	0.001
Death Attitude Investigation (DAP-R) a Means of Escape	Avoiding death	0.17	-1.20	1.54	0.806	0.12	-1.25	1.49	0.864
Acceptance of death	Fear of death	2.19	1.10	3.28	<0.001	2.20	1.11	3.29	<0.001
Neutral acceptance		Daily Spiritual Experience (DSES) b				-0.03	-0.10	0.04	0.345
R^2^ (adjusted)		0.160 (0.123)				0.164 (0.122)			

## Discussion

The study aimed to analyze medical and nursing staff's opinions and perspectives on euthanasia, identifying factors that influenced their beliefs and attitudes. Euthanasia is a complex linguistic concept, including voluntary, involuntary, active, passive, and voluntary forms, which can be confusing to health professionals [12,13]. These experts' perspectives are frequently established in this direction, which may have an impact on how they react in difficult situations related to their employment, including cases where euthanasia may be related to stroke death and transplantation [13].

Several studies have been carried out in a wide range of countries to explore health professionals' attitudes towards euthanasia, particularly physicians and nurses, both during undergraduate education and after long-term work experience. Although the current study only investigated medical and nursing staff, research on the subject of "euthanasia" has been undertaken in a number of professional or population groups, including physicians, nurses, exclusive carers or carers/relatives, medical and nursing students, and so on. The study suggests that few elderly patients support the procedure, while more patient carers, primarily doctors, approve it under specific circumstances. Medical students are generally opposed, and nursing students in Greece show a lack of knowledge and a generally opposed attitude toward legalization [14-16].

In the current study, a combined effort was made to correlate this attitude with other factors, such as attitude towards death itself, religiosity and spirituality, or specific characteristics, such as gender, age, marital status, or job position, that are specifically related to medical and nursing staff, as the literature has shown diverse results regarding the attitudes of medical and nursing personnel. According to comparable studies, the findings may differ or even coincide [17-19].

Cohen and colleagues investigated the level of acceptance of euthanasia among citizens, as well as its relationship to religious and socio-demographic factors, or between countries and religious denominations, in 47 European countries and 67,786 people. According to their findings, Western countries, even those with relevant legislation, have the highest degree of acceptance. On the contrary, it appears to have less appeal as a concept and as a process in the more eastern states, showing the acceptability gap between the West and the East, making a European solution to the issue impossible [20].

Another study of 120 physicians in the Athens region using the DSES Scale indicated an average score of 52.25, which is lower than that of the participants in the current study. Nonetheless, when euthanasia attitudes were analysed, they were revealed to be significantly related to their expertise, years of competence, and the number of patients in the terminal stage of the disease who exhausted and died in the previous 12 months (p <0.05) [9].

Limitations

The research assessed attitudes and perceptions regarding euthanasia through voluntary participation and indirect bioethical issues. However, participation was not universal, with only potential medical and nursing staff in Lasithi prefecture participating. This suggests an uncontrollable bias, but it is common in relevant research, leading to low response rates due to sampling based on convenience or convenience.

Because it is cross-sectional research, the results cannot be interpreted causally. Despite this, an attempt was made to collect data from a variety of hospitals and health institutions located geographically across a Crete prefecture with long distances between them, with the researcher making a special personal effort to obtain the best possible answer.

## Conclusions

This research investigated the opinions and perspectives of medical and nursing hospital staff regarding euthanasia and detected the factors that were for and against it. Health professionals were found to have moderate attitudes about euthanasia, with no significant difference between them, as well as moderate degrees of death and everyday spiritual experience. To enhance death-related care, it's vital to emphasize the importance of death education for medical and nursing practitioners and allied health workers. Prioritizing the patient's benefit and protection is crucial despite the influence of moral establishments, religious beliefs, and cultural, ideological, and political factors on each country's approach to the issue.

## References

[1] Beauchamp TL, Childress JF (2012). Principles of Biomedical Ethics, 7th edn.

[2] Protopapadakis Protopapadakis, Evangelos (2018) (2024). Why letting die instead of killing? Choosing active euthanasia on moral grounds. Proceedings of the XXIII World Congress of Philosophy. Proceedings of the XXIII World Congress of Philosophy.

[3] Borry P, Schotsmans P, Dierickx K (2006). Empirical research in bioethical journals. A quantitative analysis. J Med Ethics.

[4] Akdeniz M, Yardımcı B, Kavukcu E (2021). Ethical considerations at the end-of-life care. SAGE Open Med.

[5] Inbadas H, Zaman S, Whitelaw S, Clark D (2017). Declarations on euthanasia and assisted dying. Death Stud.

[6] Inbadas H, Carrasco JM, Clark D (2020). Representations of palliative care, euthanasia and assisted dying within advocacy declarations. Mortality (Abingdon).

[7] Kozamani Α (2019). Euthanasia: practices applied by the countries of the European Union. Bioethica.

[8] Kontaxakis V, Paplos KG, Havaki-Kontaxaki BJ, Ferentinos P, Kontaxaki MI, Kollias CT, Lykouras E (2009). Attitudes on euthanasia and physician-assisted suicide among medical students in Athens. Psychiatriki.

[9] Malliarou M, Tzenetidis V, Papathanasiou I, Vourdami K, Tzenetidis N, Nikolentzos A, Sarafis P (2022). Validation of the Greek version of Euthanasia Attitude Scale (EAS) in Greek medical doctors. Nurs Rep.

[10] Wong PP, Reker TG, Gesser G (1994). Death Attitude Profile-Revised: A Multidimensional Measure of Attitudes Toward Death. Death Anxiety Handbook: Research, Instrumentation, And Application.

[11] Underwood LG, Teresi JA (2002). The daily spiritual experience scale: development, theoretical description, reliability, exploratory factor analysis, and preliminary construct validity using health-related data. Ann Behav Med.

[12] Mastorakis K, Baloyiannis S (2010). The issue of euthanasia and the modern problem. Encephalos.

[13] Malliarou M, Tzenetidis V, Papathanasiou I, Vourdami K, Tzenetidis N, Nikolentzos A, Sarafis P (2022). Physicians' attitudes towards euthanasia and correlation with their spirituality. Psychiatriki.

[14] Kranidiotis G, Ropa J, Mprianas J, Kyprianou T, Nanas S (2015). Attitudes towards euthanasia among Greek intensive care unit physicians and nurses. Heart Lung.

[15] Leppert W, Gottwald L, Majkowicz M, Kazmierczak-Lukaszewicz S, Forycka M, Cialkowska-Rysz A, Kotlinska-Lemieszek A (2013). A comparison of attitudes toward euthanasia among medical students at two Polish universities. J Cancer Educ.

[16] Suen LJ, Lee HH, Morris DL (2013). Life-sustaining treatment: a comparison of the preferences of Taiwanese older adults and their family caregiver. J Nurs Res.

[17] Reis de Castro MP, Antunes GC, Pacelli Marcon LM, Andrade LS, Rucki S, Andrade VA (2016). Euthanasia and assisted suicide in western countries: a systematic review. Revista Bioética.

[18] Golestan F, Zabetian H, Zarei MJ, Jahromy FH, Kalani N, Abiri S (2019). Attitudes of students of Jahrom University of Medical Sciences toward euthanasia. J Res Med Dent Sci.

[19] Senmar M, Rafiei H, Alipour R, Yousefi F, Elikaei N, Najafi M, Bokharaei M (2016). Clinical registered nurses attitude toward euthanasia: a cross sectional study from Iran. Int J Novel Res Healthcare Nurs.

[20] Cohen J, Marcoux I, Bilsen J, Deboosere P, van der Wal G, Deliens L (2006). European public acceptance of euthanasia: socio-demographic and cultural factors associated with the acceptance of euthanasia in 33 European countries. Soc Sci Med.

